# Health-related quality-of-life of WHO grade 4 astrocytoma patients receiving alternating electrical field therapy: a prospective real-world multi-centre study

**DOI:** 10.1007/s11060-026-05631-2

**Published:** 2026-05-21

**Authors:** Desiree K. K. Wong, Jenny K. S. Pu, Lai-Fung Li, Victor K. H. Hui, Kevin K. F. Suen, Arthur C. K. Lau, Danny T. M. Chan, Michael W. Y. Lee, Tony K. T. Chan, Jason M. K. Ho, Ka-Man Cheung, Teresa P. K. Tse, Sarah S. N. Lau, Joyce S. W. Chow, Michael K. W. See, Natalie M. W. Ko, Herbert H. F. Loong, Dennis K. C. Leung, Aya El-Helali, Tai-Chung Lam, Fung-Ching Cheung, Wai-Sang Poon, Peter Y. M. Woo

**Affiliations:** 1 Department of Neurosurgery, Prince of Wales Hospital, Shatin, Hong Kong; 2 Department of Neurosurgery, Queen Mary Hospital, Pok Fu Lam, Hong Kong; 3 Department of Neurosurgery, Pamela Youde Nethersole Eastern Hospital, Chai Wan, Hong Kong; 4 Department of Neurosurgery, Princess Margaret Hospital, Kwai Chung, Hong Kong; 5 Department of Neurosurgery, Tuen Mun Hospital, Tuen Mun, Hong Kong; 6 Department of Clinical Oncology, Queen Elizabeth Hospital, Kowloon, Hong Kong; 7 Department of Neurosurgery, Queen Elizabeth Hospital, Kowloon, Hong Kong; 8 Department of Neurosurgery, Kwong Wah Hospital, Yau Ma Tei, Hong Kong; 9 Department of Clinical Oncology, The Chinese University of Hong Kong, Shatin, Hong Kong; 10 Department of Clinical Oncology, The University of Hong Kong, Pok Fu Lam, Hong Kong

**Keywords:** Alternating electric fields, Tumour treating fields, WHO grade 4 astrocytoma, Glioblastoma, Quality-of-life

## Abstract

**Purpose:**

Alternating electric fields (AEF) therapy has been shown to improve the overall survival of patients with WHO grade 4 astrocytoma. Few have explored its impact on health-related quality-of-life (HRQoL) and caregiver stress.

**Methods:**

This was a prospective, multi-centre, registry-based study of adult patients with WHO grade 4 astrocytoma who received either AEF plus temozolomide chemoradiotherapy (AEF + CRT) or CRT alone, with EORTC QLQ-C30/BN20, CSI, and HK-MoCA assessments at baseline and every three months. The primary outcome was the global QLQ-30 score at 3 months after CRT. Secondary outcomes included the QLQ-30 functional score, symptom scores and the caregiver stress index (CSI) at 3 months.

**Results:**

88 patients, 48 AEF + CRT and 40 CRT-alone, were reviewed. The mean AEF duration was 10 ± 8 months. At 3 months, AEF + CRT patients had significantly lower global QLQ-C30 scores than CRT-alone patients (44 ± 22 vs. 66 ± 20; between-group difference 22 points; *p* < 0.001), exceeding the established minimally important difference of 4–6 points for glioma. On multivariable analysis, AEF therapy (adjusted OR 0.09, 95% CI 0.006–0.7) and preoperative KPS ≥ 80 (adjusted OR 0.09, 95% CI 0.01–0.5) were associated with lower odds of high global QoL (≥ 60). Functional and symptom scores were comparable between groups, apart from higher scalp pruritus in AEF + CRT patients. Mean caregiver CSI scores remained < 7 at all time points.

**Conclusion:**

In this prospective real-world cohort of Chinese patients with WHO grade 4 astrocytoma, AEF therapy was independently associated with a reduction in global HRQoL at 3 months compared with CRT alone, while functional and symptom domains and caregiver stress remained largely stable. These data should inform shared decision-making when weighing the survival benefit of AEF against its short-term QoL burden, especially in subtropical climates where scalp-related adverse effects may be more pronounced.

**Supplementary Information:**

The online version contains supplementary material available at 10.1007/s11060-026-05631-2.

## Introduction

Glioblastoma (WHO grade 4 astrocytoma, IDH-wildtype) is the most common primary malignant brain tumour in adults. Despite standard-of-care (SOC) treatment, comprising maximal safe resection followed by concomitant temozolomide chemoradiotherapy (TMZ-CRT) and subsequent six cycles of adjuvant maintenance TMZ, the prognosis is generally poor with a median overall survival (mOS) of 13.6 to 14.6 months [1, 2]. The process of coping with the treatment is taxing, resulting in the deterioration of health-related quality of life (HRQoL) outcomes for patients and increased caregiver stress [3–5]. In this study, we use ‘World Health Organisation (WHO) Grade 4 astrocytoma’ as an umbrella term encompassing both glioblastoma, IDH-wildtype, central nervous systen (CNS) WHO Grade 4 astrocytoma, IDH-mutant, CNS WHO Grade 4 astrocytoma, reflecting the 2016 4th edition of the WHO Classification of Tumours of the CNS (CNS4) framework during most of the recruitment period.

In recent years, alternating electrical field (AEF) therapy, also known as tumour-treating fields, has been introduced as an adjuvant treatment in addition to first-line SOC and is incorporated into several clinical practice guidelines [6]. AEF therapy is a non-invasive regional treatment modality where scalp transducers deliver low intensity (1–3 V/cm), intermediate frequency (200 kHz) electric fields to the post-tumour resection region. The biophysical force exerted by alternating electric fields inhibits tumour cell growth and mitosis, leading to apoptosis [7]. The EF-14 study, a multi-centre randomized-controlled trial demonstrated the efficacy of AEF therapy with adjuvant TMZ for newly-diagnosed glioblastoma patients, whereby mOS was increased from 16.0 months to 20.9 months [8]. Several studies demonstrated its therapeutic benefit, including a propensity-score matched study of Chinese patients, and a meta-analysis confirmed its role [9, 10]. 

HRQoL is an important consideration for WHO grade 4 astrocytoma patients receiving AEF therapy, as it may directly affect treatment compliance, an established independent factor for OS [11, 12]. The EF-14 study determined that acceptable AEF device utilization required a minimal mean monthly treatment time of 75%, although other studies revealed that a lower threshold of 60% was still beneficial [8, 12, 13]. AEF therapy requires patients to carry a cumbersome device for a substantial portion of the day, undergo frequent hair clipping, and regular scalp array application by their caregivers. We theorize that such treatment commitments may explain why a minority of patients receive AEF therapy, and that only 30% of neuro-oncologists consider it a definitive component of first-line SOC [14]. Few studies have explored the effect of AEF on the QoL of these patients in a real-world setting, and most focused on Western populations [15–17]. None have evaluated caregiver psychological stress. To address these issues, we prospectively reviewed data from Chinese patients who received AEF therapy.

## Materials and methods

### Patient selection and data collection

This was a territory-wide, multicentre prospective study of histologically-confirmed, newly-diagnosed adult (*≥* 22 years) patients with WHO grade 4 astrocytoma who received adjuvant AEF therapy with SOC from 1 January 2017 to 1 October 2023. The study was approved by the Hong Kong Hospital Authority (HA) institutional review board (reference number: UW19-626) and followed the Declaration of Helsinki and Good Clinical Practice guidelines. Informed consent was obtained from patients, their caregivers and legal representatives. Patients were grouped according to whether they received CRT with AEF therapy (AEF + CRT group) or CRT-alone (control group), based on treatment received as recorded in the Hong Kong Glioma Registry. Treatment allocation was non-randomized and determined by regulatory availability, patient preference, and financial accessibility. AEF therapy (Optune™, Novocure GmbH, Root, Switzerland) was first introduced to Hong Kong in January 2019, with patients either self-financing their treatment or receiving government subsidies after means testing.

The diagnosis of WHO grade 4 astrocytoma was established in accordance with the 4th edition of WHO Classification of Tumours of the CNS. All patients underwent maximal safe resection and completed standard TMZ CRT [18]. The standard TMZ dose was 75 mg/m^2^/day for 6 weeks, administered concomitantly with RT of 60 Gy (2 Gy in 30 fractions), followed by maintenance chemotherapy with TMZ 150–200 mg/m^2^/day for five days every four weeks for a minimum of six cycles [2]. Patients received AEF therapy within one month after completion of CRT and were monitored monthly for adverse effects and compliance, with data extracted by a registered nurse who performed monthly home visits. Patients were also clinically reviewed at their respective centres each month and monitored for adverse effects. Patients were excluded if they: did not complete TMZ-CRT; had fewer than 3 months of follow-up; did not undergo maximal safe resection; had cerebellar tumours; had received prior radiotherapy; had a prior histopathological diagnosis of a lower-grade glioma; had a concomitant disabling condition precluding a preoperative Karnofsky Performance Status (KPS) ≥ 80; or, for the AEF group, had shorter than 1 month of AEF use. Clinical data were retrieved from the Hong Kong Glioma Registry, a prospectively collected population-level central database of adult patients with histologically confirmed glioma [1]. Data were characterized into patient (age, sex, preoperative KPS), tumour (tumour location, isocitrate dehydrogenase-1 (*IDH-1*) mutation status, and methylguanine methyltransferase promoter methylation (p*MGMT)* status), and treatment-related factors. Extent of resection (EOR) was determined by reviewing postoperative day-one magnetic resonance imaging (MRI) scans according to the response assessment in neuro-oncology (RANO) resect criteria [19] or by the neurosurgeon’s assessment documented in operation records. EOR was categorized into gross total resection (GTR), which was defined as no measurable residual enhancing tumour on postoperative day-one gadolinium-enhanced MRI per the RANO-resect criteria; subtotal resection (STR), which was defined as any measurable residual enhancement on postoperative MRI; or biopsy, where no resective procedure was attempted. Under the subsequently published 2021 WHO CNS Classification (CNS5) [20], 83% of the cohort (IDH-wildtype) would be reclassified as ‘Glioblastoma, IDH-wildtype, CNS WHO grade 4’, while the remaining 17% (IDH-mutant) correspond to ‘Astrocytoma, IDH-mutant, CNS WHO grade 4’. However, all diagnoses made were based on the 2016 CNS4 framework during the recruitment period of this study. Therefore, in this study, we use ‘WHO Grade 4 astrocytoma’ as an umbrella term encompassing both glioblastoma, IDH-wildtype, CNS WHO Grade 4 and astrocytoma, IDH-mutant, CNS WHO Grade 4.

### Assessment of HRQoL

All patients were assessed at regular three-month intervals within two weeks of completion of concomitant TMZ CRT, and for up to 12 months, using validated HRQoL questionnaires. The European Organisation for Research and Treatment of Cancer (EORTC) core quality of life questionnaire version 3.0 (QLQ-C30) and the additional module for brain cancer (QLQ-BN20) were adopted [21, 22], with 30 questions covering various HRQoL domains. There are five functional scales (physical, role, emotional, cognitive and social functioning), nine symptom scales (fatigue, nausea/vomiting, pain, dyspnea, insomnia, appetite loss, constipation, diarrhea and financial difficulties), and global health status [21]. The QLQ-BN20 is specific for brain cancer patients and comprises 20 questions representing 11 symptom scales (future uncertainty, visual disturbance, motor dysfunction, communication deficit, headaches, seizures, drowsiness, itchy skin, hair loss, lower limb weakness, and bladder control) [22]. Patient responses were converted to linear scales ranging from 0 to 100, with a higher score referring to a higher global health score or functioning score. In contrast, a higher score on the symptoms scale indicates greater severity [21]. These questionnaires have been extensively validated as reliable measures of cancer and glioblastoma HRQoL in diverse clinical settings [23–25]. The English or validated Chinese translated versions of the above questionnaires were utilized [26, 27]. Longitudinal analyses were conducted on an observed-case basis at each time point, recognizing that missing data points were predominantly due to clinical deterioration or death.

### Assessment of caregiver stress

For AEF therapy patients, primary caregivers (responsible for array application) were assessed by the Caregiver Strain Index (CSI) at the aforementioned time intervals [28, 29]. The CSI is a validated 12-question survey exploring the physical, emotional, financial, and social burdens of caregivers [30, 31]. This index is frequently used to assess caregiver strain among advanced cancer patients, and a score *≥* 7 indicates high stress [31]. The English or the validated Chinese version of the CSI was utilized [28]. 

### Assessment of cognitive scores

Cognitive function was assessed using the Hong Kong version of the Montreal Cognitive Assessment (HK-MoCA), a validated 30-point cognitive screening instrument adapted for Cantonese-speaking Chinese adults. The HK-MoCA has been validated against expert DSM-IV criteria for dementia and mild cognitive impairment, with a sensitivity of 92.8% and specificity of 73.5% at the recommended cutoff of < 22 (adjusted for education level) for the detection of cognitive impairment in Hong Kong Chinese older adults [32]. A validation study confirmed its high intra- and inter-rater reliability as well as good internal consistency in Chinese adults, with an area under the ROC curve of 0.92 (95% CI 0.88–0.966) [32]. 

To ensure consistency across centres, a research coordinator received specialized training for the administration of the HK-MoCA, HR-QoL instruments, and the CSI. Training was provided by a senior occupational therapist with more than 10 years of experience in neuro-rehabilitation. Investigators responsible for multivariate analysis were not involved in the prescription of AEF therapy to individual patients, and the treating clinical team was blinded to the HK-MoCA, CSI, and QoL outcomes.

### Statistical analysis

The primary endpoint was the EORTC QLQ-C30 global score at three months. Secondary endpoints included the QLQ-BN20 score, the CSI for caregivers of AEF + CRT patients, and HK-MoCA scores, at three-monthly intervals. Descriptive statistics were used for demographic data. Paired comparisons over time were evaluated using the Wilcoxon signed-rank test for HRQoL, CSI, and HK-MoCA scores. Pearson’s chi-squared test (categorical variables) was performed for between-group differences. Continuous variables between independent groups were assessed for normality using the Shapiro-Wilk test. Since the HRQoL data were found to be non-normally distributed (for 3-month Global EORTC scores between groups, W = 0.9, *p* < 0.01), group comparisons for primary HRQoL outcomes were performed using the Mann-Whitney U test. Multivariate logistic regression was performed for the primary endpoint at 3 months, and was restricted to patients with available 3-month global scores; no imputation was performed for missing HRQoL data. As there are no standardized EORTC QLQ-30 cut-off scores for neuro-oncology patients, the cohort’s median EORTC global score of 60 was utilized as the cut-off to determine patients with higher QoL, similar to the EORTC QLQ-30 reference value for global QoL (61.3, for all cancer patients) [33]. Sample size and power considerations were determined based on the primary HRQoL endpoint of EORTC QLQ-C30 Global score at three months. To detect a clinically meaningful between-group difference of 10 points [34], a power analysis was performed. Assuming a pooled standard deviation of 21 points based on secondary HRQoL analyses from the EF-14 trial [35] an alpha of 0.05, and 80% power, a minimum of 35 patients per treatment arm was required. To adjust for confounding, significant factors identified from univariate logistic regression were subject to multivariate regression to determine independent predictors for QoL. A p-value ≤ 0.05 was considered significant. All analyses were performed on R (R Core Team, 2021).

## Results

**Fig. 1:**
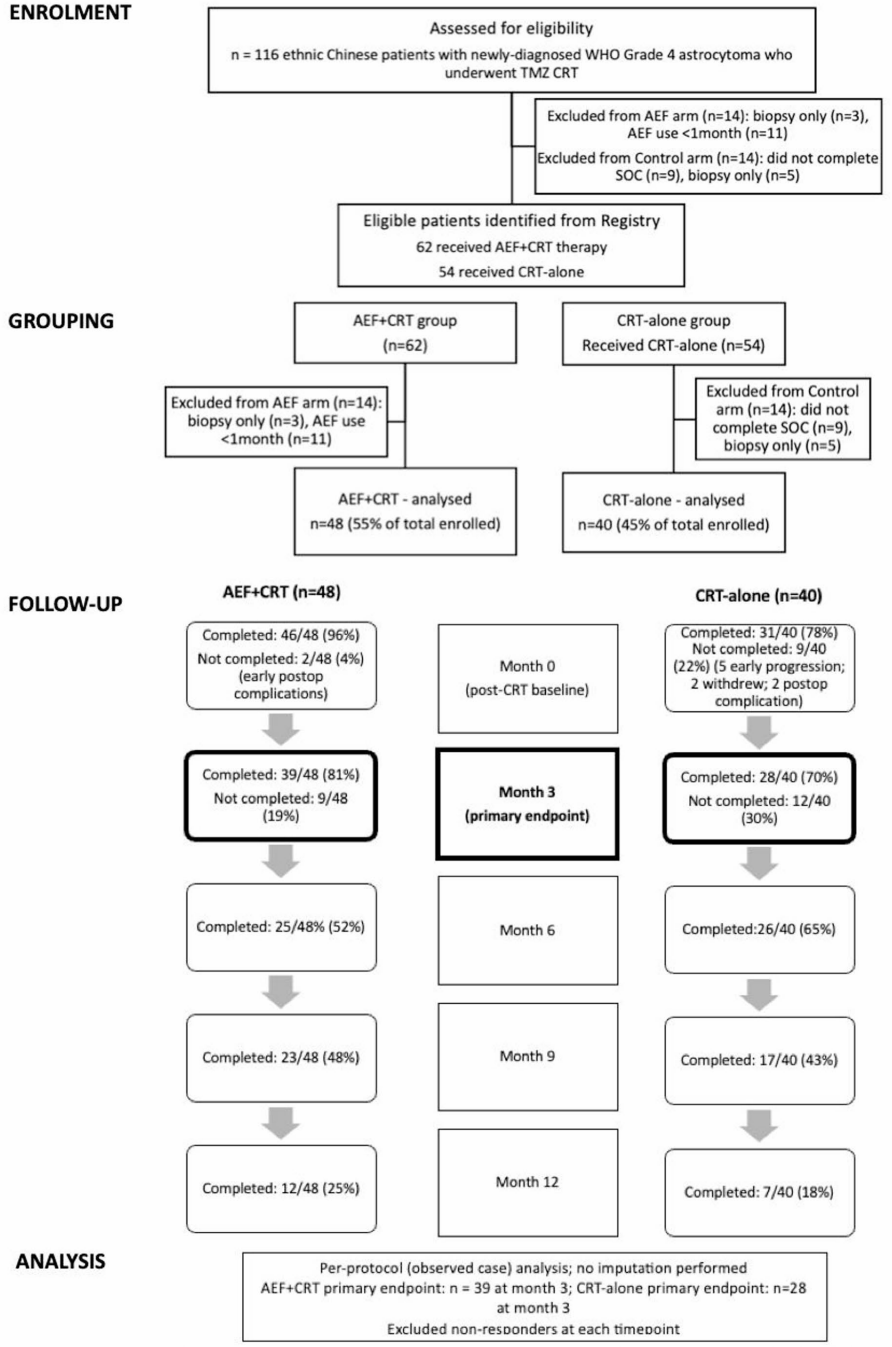
CONSORT diagram of AEF+CRT and CRT-alone control group patients. AEF: alternating electric fields. CRT: chemoradiotherapy

116 ethnic Chinese patients with newly-diagnosed WHO grade 4 astrocytoma underwent TMZ CRT during the study period. 62 patients received AEF therapy, but 14 were excluded as three underwent a tumour biopsy only and 11 had AEF therapy < 1 month. 54 patients received CRT alone, but 14 were excluded as 9 did not complete SOC, 5 had a biopsy only. The final analysis included 88 patients; 48 (55%) patients in the AEF + CRT group, and 40 (45%) in the CRT-alone control group (Fig. 1).

Mean age by group (AEF + CRT: 54 ± 13 years; CRT-alone: 52 ± 13 years; *p* = 0.41), sex distribution by group (AEF + CRT: 60% male; CRT-alone: 53% male; *p* = 0.46), preoperative KPS ≥ 80 by group (AEF + CRT: 73% [35/48]; CRT-alone: 62% [25/40]; *p* = 0.36), *IDH-1* mutation rate by group (AEF + CRT: 10% [5/48]; CRT-alone: 25% [10/40]; *p* = 0.07), *pMGMT* methylation by group (AEF + CRT: 40% [19/48]; CRT-alone: 58% [23/40]; *p* = 0.12) were comparable. There was a statistically significant difference in tumour location (frontal lobe: AEF + CRT 33% [16/48] vs. CRT-alone 48% [19/40]; *p* = 0.04). GTR was achieved in 44% (39/88) of patients and was similar for both groups. The mean duration of AEF use was 10 ± 8 months. Other patient, tumour, and treatment factors were comparable b (Table 1). The mean follow-up duration was 29 ± 16 months for the AEF + CRT group, and 36 ± 31 months for the CRT-alone group (Mann-Whitney U test, *p* = 0.18). The mOS of the AEF + CRT group was 25.1 months, and the mOS of the CRT-alone group was 27.6 (Cox regression test, *p* = 0.88).

**Table 1:** Patient, tumour and treatment characteristics of AEF + CRT patients and CRT- alone patients

	AEF + CRT group *n* = 48 (%)	CRT-alone group *n* = 40 (%)	*p*-value
**Patient factors**			
Age at diagnosis, years, mean ± SD	54 ± 13	52 ± 13	0.41^*^
Male	29 (60)	21 (53)	0.46^#^
Preop KPS ≥ 80	35 (73)	25 (62)	0.36^#^
Follow-up, months, mean ± SD	29 ± 16	36 ± 31	0.20^*^
**Tumour factors**			
Location			
Frontal	16 (33)	19 (48)	0.04^#^^
Parietal	6 (13)	10 (25)	
Temporal	15 (31)	8 (20%)	
Occipital	2 (4%)	3 (7%)	
Multifocal	5 (11)	0	
Central^‡^	4 (8%)	0	
Infratentorial	0	0	
* IDH-1* status^**†**^			
Mutant	5 (10)	10 (25)	0.07^#^
p*MGMT* status^**†**^			
Methylated	19 (40)	23 (58)	0.12^#^
**Treatment factors**			
Extent of resection			
GTR	21 (44)	18 (45)	0.91^#^
STR	27 (56)	22 (55)	

^**‡**^ Insula, basal ganglia or thalamus. ^**†**^10% were missing. *Wilcoxon test. ^#^chi-squared test . CRT: chemoradiotherapy. KPS: Karnofsky Performance Scale. *IDH-1*: Isocitrate dehydrogenase -1. p*MGMT*: promoter region of O6-methylguanine-methyltransferase. GTR: Gross Total Resection. STR: Subtotal Resection. ^ p = < 0.05

### Quality-of-life analysis

Questionnaire response rates for the EORTC QLQ-30 and BN-20 HRQoL surveys at 0, 3, 6, 9, 12 months were 96% (46/48), 81% (39/48), 52% (25/48), 48% (23/48), 25% (12/48) among AEF + CRT patients and 78% (31/40), 70% (28/40), 65% (26/40), 43% (17/40), 18% (7/40) for CRT-alone patients respectively. There was no significant difference in the response rates between the groups (chi-squared test, *p* = 0.25).

### EORTC global scores

Among the 67 patients who completed the 3-month QLQ-C30 global assessment, 28 (42%) had high global QoL (≥ 60) and 39 (58%) had low QoL (< 60). AEF + CRT patients had a significantly lower mean global HRQoL score (44 ± 22) compared with CRT-alone patients (66 ± 20) (Mann-Whitney U = 264, *P* < 0.001) at 3 months, representing a between-group difference of 22 points — more than twice the established minimally important difference (MID) of 4–6 points for glioma patients [34]. Multivariate analysis identified AEF use (adjusted OR 0.09, 95% CI [0.006, 0.7]) and preoperative KPS ≥ 80 (adjusted OR 0.09, 95% CI [0.01–0.5]) as significant independent factors associated with lower odds of high global QoL at 3 months (Table 2). Patients with preoperative KPS ≥ 80 had higher baseline (post-CRT) global scores than those with KPS < 80, however were more likely to fall below the ≥ 60 global QoL threshold at 3 months. Global scores did not change over time for the AEF + CRT group (Wilcoxon signed-rank test, *p* = 0.53).

**Table 2:** Univariate and multivariate analysis for factors predicting high global QoL (≥60) at 3 months

	High Global QoL (≥ 60) *n* = 28 (%)	Low Global QoL (< 60) *n* = 39 (%)	Univariate analysis	Multivariate analysis
			OR (95% CI)	Adjusted OR (95% CI)
**Patient factors**				
Age ≥ 65	4 (14)	6 (15)	0.93 (0.21–3.70)	
Gender (male)	12 (43)	24 (62)	0.48 (0.17–1.32)	
Preop KPS ≥ 80	15 (54)	30 (77)	0.35 (0.12-1.00)	0.09 (0.0060-0.70)^
**Tumour factors**				
* IDH-1* wildtype	19 (68)	31 (79)	2.24 (0.61–8.83)	
p*MGMT* methylated	17 (61)	16 (41)	2.00 (0.70–5.81)	
Location – frontal	12 (43)	14 (36)	1.30 (0.40–4.32)	
Laterality - right	14 (50)	25 (64)	0.49 (0.17–1.30)	
**Treatment factors**				
Gross total resection	10 (36)	18 (46)	0.65 (0.23–1.80)	
AEF therapy	10 (36)	29 (74)	0.20 (0.07–0.56)	0.09 (0.01–0.52)^^^

CRT: chemoradiotherapy. KPS: Karnofsky Performance Scale.* IDH-1*: isocitrate dehydrogenase-1. p*MGMT*: promoter region of O-6-methylguanine- methyltransferase. GTR: gross total resection. AEF: alternating electric fields. ^ p =<0.05

### EORTC functional scores

**Fig. 2:**
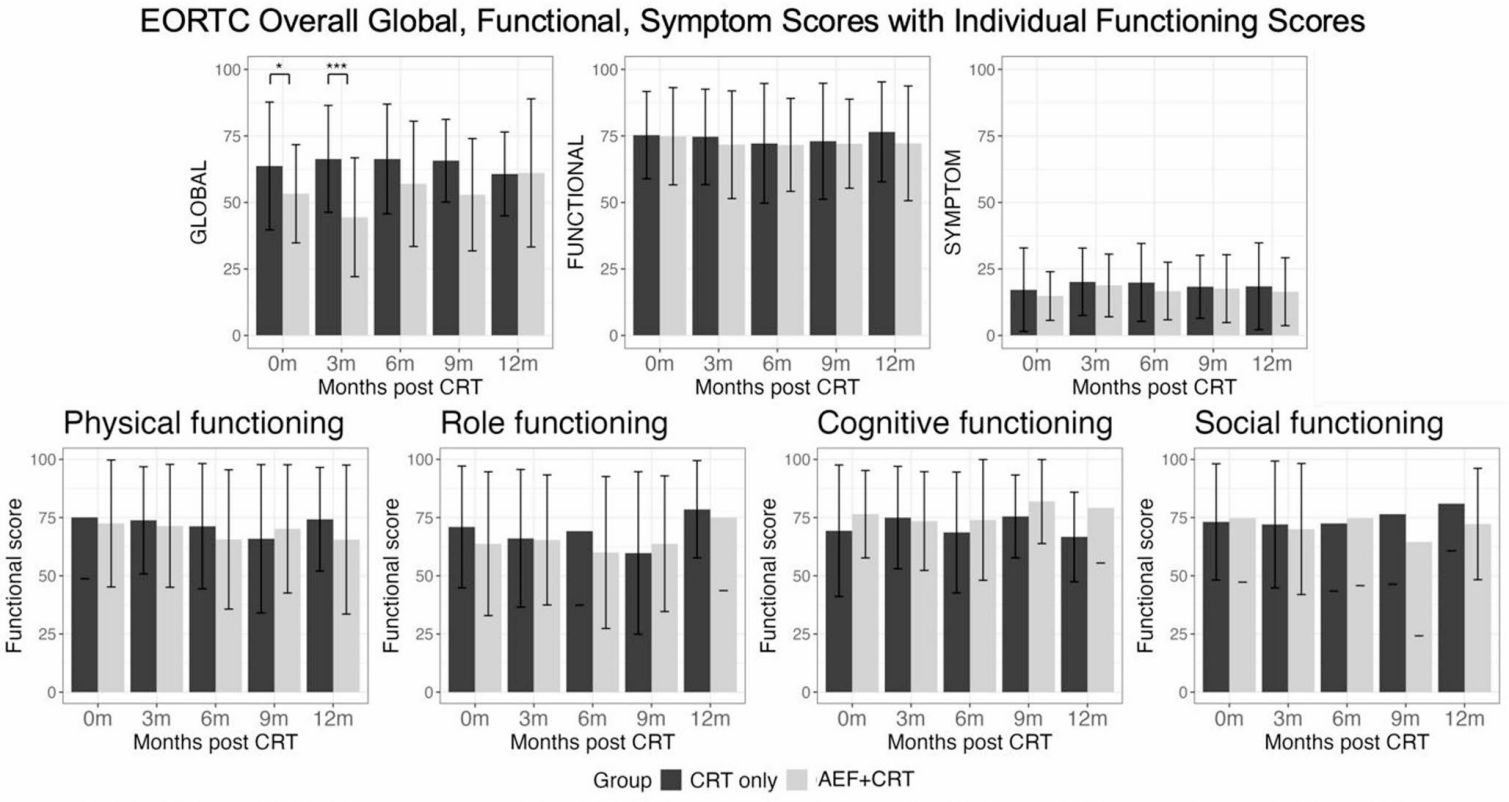
Graph showing overall EORTC scores for Global, Functional and Symptom scores, and Individual Functional Scores over time for AEF+CRT and CRT-alone control groups

There was no significant change in functional scores for the AEF + CRT group over time (paired t-test, *p* = 0.28). Both AEF + CRT and CRT-alone groups had similar scores across all functional domains (Wilcoxon signed-rank test, *p* = 0.30) (Fig. 2).

### EORTC symptom scores

**Fig. 3:**
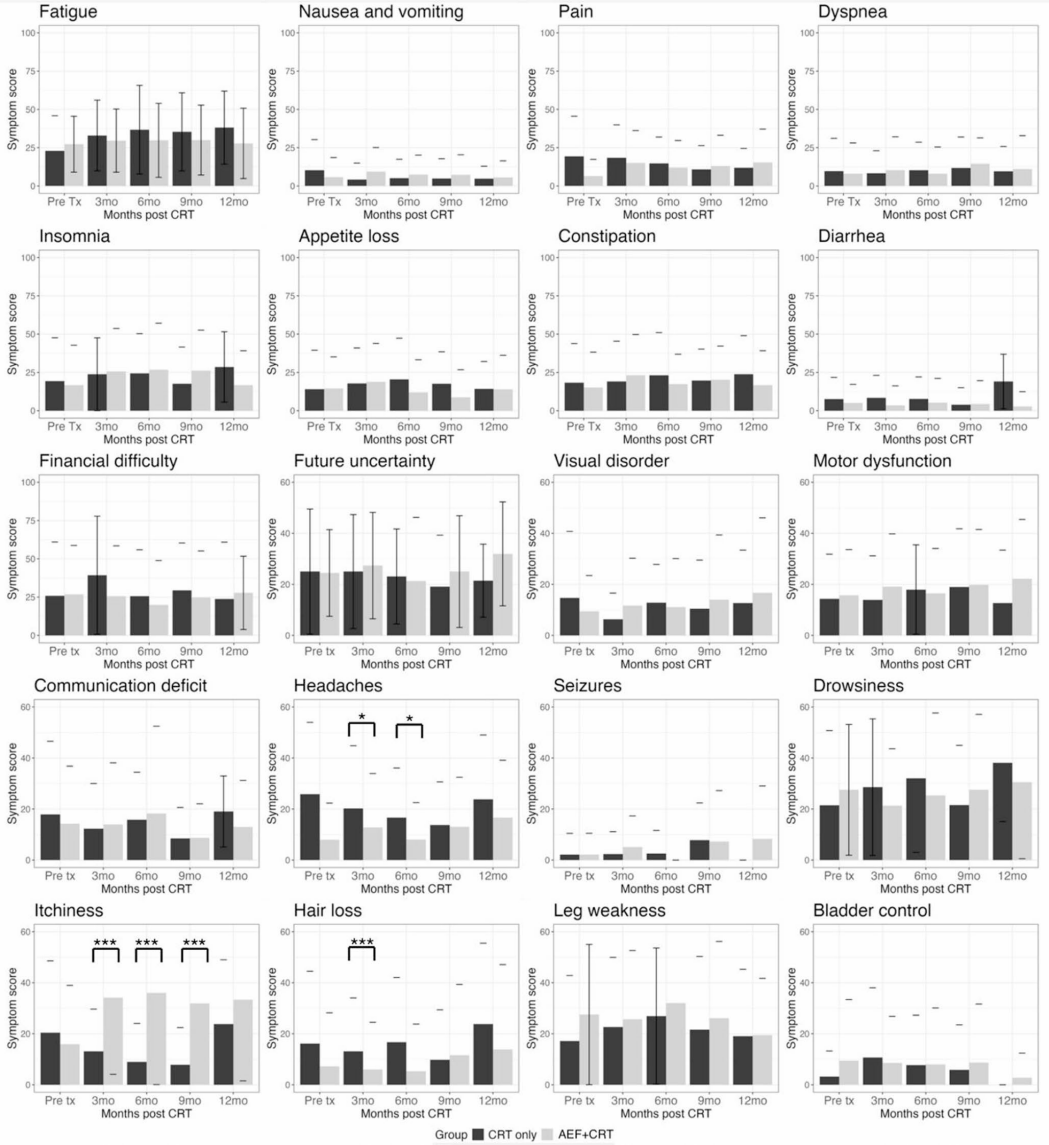
Graph showing individual QLQ-30 and BN20 symptom score comparison for AEF+CRT and CRT only groups

The overall symptom scores for the AEF + CRT group remained comparable over time (Fig. 2), from a pretreatment mean overall symptom score of 15 ± 9 to 19 ± 12 at 3 months (Wilcoxon signed-rank test, *p* = 0.59), and were comparable to CRT-alone patients (Wilcoxon signed-rank test, *p* = 0.33). For individual symptom score comparisons, general cancer QLQ-30 symptom scores were similar between AEF + CRT and CRT-alone patients (Fig. 3). Fatigue scores were the highest for each group. The AEF + CRT group had a mean 3-month score of 29 ± 21, and the CRT-alone group had a score of 33 ± 23 (Mann-Whitney U test, *p* = 0.86). For the BN-20 questionnaire brain-cancer specific symptoms, AEF + CRT patients experienced significantly higher scalp pruritus scores, with a mean score of 34 ± 30 at 3 months compared to the CRT-alone group that had a mean score of 13 ± 17 (Mann-Whitney U test, *p* < 0.001). For headache and hair loss, CRT-alone group patients had higher scores (Mann-Whitney U test: headache, *p* = 0.02; hair loss, *p* < 0.01) than AEF + CRT patients. Other BN20 symptoms were comparable (Mann-Whitney U test, *p* > 0.05) (Fig. 2).

### Caregiver stress

The CSI for patients who received AEF + CRT did not increase with time (paired t-test, *p* = 0.72). The baseline post-CRT CSI score was 5.3 ± 3.5, 4.8 ± 3.7 at 3 months, and 4.0 ± 3.9 at 6 months. The CSI was significantly reduced by nine months, with a mean CSI score of 2.8 ± 3.5 (Wilcoxon signed-rank test, p = < 0.01). The mean CSI score remained below 7 throughout the study.

### HK-MoCA scores for cognitive assessment

The AEF + CRT group had a comparable mean MoCA score (20.8 ± 8.2) to the CRT-alone group (17.2 ± 6.3) at post-CRT baseline (Mann-Whitney U test, *p* = 0.06). By three months, the mean MoCA score of the AEF + CRT group (20.2 ± 8.3) became significantly higher than the CRT-alone group (13.4 ± 8.4) (Mann-Whitney U test, *p* = 0.02). Post-hoc Spearman correlation analysis between 3-month HK-MoCA scores and mean monthly AEF compliance percentage in the AEF + CRT group was not significant (Spearman *r* = 0.18, *p* = 0.24).

## Discussion

AEF therapy, in addition to SOC treatment for patients with WHO grade 4 astrocytoma, is a recognized first-line option and is recommended by several clinical practice guidelines from the American Society of Clinical Oncology, the Society for Neuro-Oncology, and the Chinese Brain Cancer Association [36–38]. A meta-analysis of 10 independent studies showed a pooled mOS of 22.6 months with AEF + CRT, compared with 17.4 months for patients who received only CRT [9]. A multicentre propensity score-matched study of real-world experience demonstrated an increase in mOS of 8.5 months [12]. Despite the survival benefits of AEF therapy, only 10% of patients receive such treatment [14]. Barriers include limited understanding of the mechanism of AEF therapy, the prohibitive costs of treatment, or its perceived impact on HRQoL [12, 39, 40]. The latter two factors are crucial for public policy makers deciding whether new cancer therapies should be subsidized to meet societal expectations, particularly in regions where universal healthcare is available [12]. Given the short life expectancy of patients, issues concerning HRQoL become crucial in deciding whether to embark on non-curative therapy that demands considerable commitment by patients and caregivers alike in return for modest gains in survival [41]. 

Several secondary analyses of the EF-14 RCT concluded that AEF therapy did not influence patient HRQoL compared with control group patients [35–43]. However, in a highly-controlled trial context, potential discrepancies may exist when evaluating the HRQoL of subjects compared to real-world observations [44]. It is therefore important to conduct independent post-approval studies, of which only three were performed [15–17]. Two studies concluded that AEF therapy did not significantly reduce patient HRQoL, however they were both single-centre with fewer than 90 patients interviewed [16, 17]. A large self-administered EuroQoL EQ-5D-5 L questionnaire mail-in survey of 1,106 glioblastoma patients who received AEF observed that longer therapy durations were associated with improved mobility and self-care [15]. But data was only collected at a single time point that was not fixed, and there was no clear review of the patient’s oncologic treatment [15]. Since these studies had no control comparison group, factors such as EOR or p*MGMT* methylation status, known to have a significant impact on survival outcomes and thereby HRQoL, may have confounded these findings [15]. Finally, EORTC assessment instruments are a widely reported standard metric for HRQoL when evaluating therapeutic cancer interventions. The EuroQoL questionnaire is neither cancer nor brain tumour specific [35, 45, 46]. Given these gaps, we believed there was a need for a real-world, multi-centre study using validated EORTC HRQoL tools that included a control group. As the nature of this therapy depends heavily on the assistance of caregivers to apply the adhesive arrays and manage device malfunctions, it was also vital to assess caregiver stress.

At the primary endpoint of 3 months post-CRT, AEF + CRT patients had significantly lower global HRQoL scores compared to CRT-alone patients (44 ± 22 vs. 66 ± 20, Mann-Whitney U test, *p* < 0.001), with a between-group difference of 22 points, which is more than twice the established MID of 4–6 points in glioma patients. AEF therapy and preoperative KPS ≥ 80 were independent predictors of lower odds of high global QoL at this time point. However, there was no significant deterioration in global QoL over time within the AEF + CRT group, and functional and symptom scores were comparable between groups. Given the non-randomized, registry-based study design, these results should be interpreted as associations rather than definitive causal effects, despite adjustment for key prognostic covariates.

The central clinical question is whether the 22-point reduction in global HRQoL at 3 months observed in our AEF + CRT cohort is an acceptable cost for the documented survival benefit of this therapy. In the EF-14 randomized controlled trial, AEF therapy combined with adjuvant TMZ improved median OS from 16.0 to 20.9 months compared with TMZ alone, a gain of 4.9 months [8]. This survival benefit has been corroborated in our own multicenter propensity-score adjusted analysis of the same cohort [10], which demonstrated a comparable improvement in mOS (8.5 months) in Chinese patients. The EF-14 secondary HRQoL analysis revealed no deterioration in global QoL with AEF therapy [35]. Yet, our real-world data suggest that adherence to an intensive AEF regimen outside a trial context, particularly in a subtropical climate, was associated with a reduction in global QoL at the 3-month post-CRT timepoint. Crucially, global QoL scores in the AEF + CRT group did not deteriorate further over time, suggesting that the initial burden of adaptation to AEF therapy does not compound with continued treatment. Patients, families, and clinicians must weigh this context-specific, time-limited QoL burden against a 5-month survival benefit. A qualitative study of 31 AEF patients reported considerable adjustments to daily living, especially regarding the treatment’s visibility, device weight, and the management of scalp dermatitis [41]. Hong Kong is a south-east coastal city in China with a humid and warm subtropical climate, with an annual mean temperature of 25˚C-36˚C, and a mean relative humidity of 82% in the summer months [47]. We believe that wearing the AEF therapy scalp electrodes along with a wig or headwear in this climate could have contributed to the higher scalp pruritus scores. Coupled with the need to have the device activated for at least 60–75% of the day in order to attain a survival benefit, may explain why 77% of AEF therapy patients residing in Hong Kong experienced scalp dermatitis [9, 10, 48]. Mitigation of scalp dermatitis includes the application of topical corticosteroids, hydrogels, moisturizers, or anti-pruritic medication [49]. Severe skin ulceration or erosions may require complete interruption of AEF treatment or the adjustment of scalp array positions away from areas of significant skin loss [48].

Apart from scalp pruritus, there were no significant differences in the physical, social, and role functioning scores compared with CRT-alone patients, corroborating the findings from previous studies [35, 42]. Conversely, CRT-alone patients had more brain cancer-related symptoms such as headache, as denoted by the BN20 questionnaire, which could be related to earlier disease progression [8, 10]. In addition, AEF therapy patients were unlikely to perceive hair loss as an HRQoL adverse effect, since they have already learnt to accept regular scalp shaving as an integral part of treatment.

Preoperative KPS ≥ 80 was an unexpected independent predictor of lower 3-month HRQoL, and several factors may account for this finding. Patients with higher baseline function often begin from a near-normal level, so treatment-related decline after surgery, CRT, or AEF may be disproportionately apparent on self-reported scales such as the EORTC questionnaire. In addition, patients who are cognitively intact and socially active may experience a greater psychological burden when confronted with functional loss and treatment-related disruption. Psychological distress is common in brain tumour populations, with studies reporting substantial rates of depression, anxiety, and fear of progression [50, 51]. Higher-functioning patients may also be more attuned to subtle changes in daily functioning and therefore report impairment more readily on HRQoL instruments. This association may partly reflect our dichotomization at a global QoL threshold of ≥ 60, as patients starting from a high baseline functioning are likely to cross this cut-off after treatment-related decline. For patients with high KPS ≥ 80, who appear particularly at risk of global QoL reduction (adjusted OR 0.09), early psychosocial support and proactive scalp care may mitigate this burden and should be integrated into standard AEF clinical management pathways.

With regards to caregiver stress, it has been well-documented that caregivers of brain cancer patients experience high psychological distress, especially when the prognosis is dismal and when cognitive and physical deterioration frequently accompanies disease progression [4, 41]. Since the regular use of AEF scalp arrays requires attentive care from a committed caregiver, we hypothesized that stress levels would be significantly elevated. However, this was not noted and may reflect that AEF treatment was not particularly burdensome for caregivers, unlike our observations for patients. Perceiving cancer treatment as beneficial has been observed to reduce caregiver stress, especially among parents of children with malignancy [52]. This positive perception can lead to decreased caregiver fatigue, improved sleep, and a more optimistic outlook, which, in turn, builds family resilience and lowers caregiver anxiety [53]. Since AEF therapy in Hong Kong included regular monthly nurse home visits and a telephone hotline staffed by healthcare professionals, caregivers may have felt less distressed when such support mechanisms were available.

There are several study limitations. This was a non-randomized prospective study without subgroup matching, which may have introduced selection bias and heterogeneity. We adopted a per-protocol (PP) approach rather than an intention-to-treat (ITT) analysis for HRQoL outcomes. There was missing data, with questionnaire response rates of 52% (25/48) at six months for the AEF + CRT group, which could be due to rapid disease progression or fatigue due to second-line therapy. While an ITT approach can preserve randomisation, its application in longitudinal HRQoL research for high-grade glioma patients may paradoxically overestimate QoL by failing to account for the rapid deterioration that precedes study dropout. In both groups, non-responders at later time points tended to have worse clinical status, so the observed HRQoL trajectories could have overestimated the average HRQoL of the treated population. This bias was more pronounced in the CRT-alone group, where early non-responders had lower preoperative KPS. Another limitation was that postoperative functional performance data was not collected. Surgical resection can limit a patient’s functional status and fitness to undergo CRT and AEF. Postoperative KPS has been shown to be a superior predictive factor compared to preoperative KPS for OS [54]. A third study limitation was utilizing the 3-month endpoint for HRQoL outcomes. Presenting longitudinal data for between-group comparisons at 6, 9 and 12 months would have provided insight into the HRQoL outcomes over time, however, the progressively declining sample size at these time points (52% and 65% response rates at 6 months for the AEF and control groups respectively, falling to 25% and 18% at 12 months) precluded statistically valid between-group comparisons . Additionally, there was no comparison of recurrence requiring repeat resection or second-line therapies , which may have affected QoL scores. Moreover, individual hospital centre was not included as a covariate in the multivariate model, due to small per-centre sample sizes exposing the study to potential centre bias. A formal mixed-effects model with a random centre effect could be explored in future studies with larger multicentre samples. Another recognized limitation was that 83% of the cohort carried IDH-wildtype tumours, which under the 2021 WHO CNS Classification are designated ‘Glioblastoma, IDH-wildtype, CNS WHO grade 4’, while the remaining 17% would be reclassified as ‘Astrocytoma, IDH-mutant, CNS WHO grade 4’, a biologically and prognostically distinct entity with a significantly better mOS [20]. In the present study, *IDH-1* mutation status was not an independent predictor of global QoL at 3 months. Nevertheless, the inclusion of a small proportion of IDH-mutant cases may have introduced prognostic heterogeneity, and a formal IDH-stratified QoL subgroup analysis was precluded by the limited number of IDH-mutant patients. Future studies should prospectively stratify by IDH status to better characterize its influence on HRQoL outcomes. Finally, more nuanced patient-reported outcome measures (PROMs) were not assessed. The addition of AEF therapy brings visibility to a patient’s identity and disease journey. The addition of qualitative patient-focused interviews with open-ended questions could have provided further insight for patients undergoing AEF therapy.

This is the first study to evaluate the impact of AEF therapy on HRQoL in Chinese patients with WHO Grade 4 astrocytoma. AEF was generally tolerable, with functional and symptom domain scores remaining stable over time, but AEF + CRT patients had significantly lower global scores than CRT-alone patients at the 3-month primary endpoint. Since scalp pruritus was the commonest adverse effect associated with AEF therapy, patients residing in subtropical climates require more attentive scalp care.

## Supplementary Information

Below is the link to the electronic supplementary material.

Supplementary Material 1

Supplementary Material 2

## Data Availability

The datasets analysed during the current study are available from the corresponding author on reasonable request.

## Abbreviations

AEF: Alternating electric fields
aHR: Adjusted hazard ratio
ANOVA: Analysis of variance
CI: Confidence interval
CNS: Central nervous system
CRT: Chemoradiotherapy
EOR: Extent of resection
GTR: Gross total resection
HA: Hospital Authority
HRQoL: Health-related quality of life
*IDH-1*: Isocitrate dehydrogenase-1
IQR: Interquartile range
KPS: Karnofsky Performance Status
MRI: Magnetic resonance imaging
NCDB: National Cancer Data Base
OS: Overall survival
PFS: Progression-free survival
pMGMT: Methylguanine-methyltransferase promoter
RCT: Randomized-controlled trial
RTOG: Radiation Therapy Oncology Group
SD: Standard deviation
SNO: Society for Neuro-oncology
SOC: Standard-of-care [17
STR: Subtotal resection
TMZ: Temozolomide
TTFields: Tumour treating fields
WHO: World Health Organization
