# Supplementary figures and images for: Health-related quality-of-life of WHO grade 4 astrocytoma patients receiving alternating electrical field therapy: a prospective real-world multi-centre study
Source: J Neurooncol. 2026 May 21;178(1):16. doi: 10.1007/s11060-026-05631-2 (PMC13194222; doi:10.1007/s11060-026-05631-2)

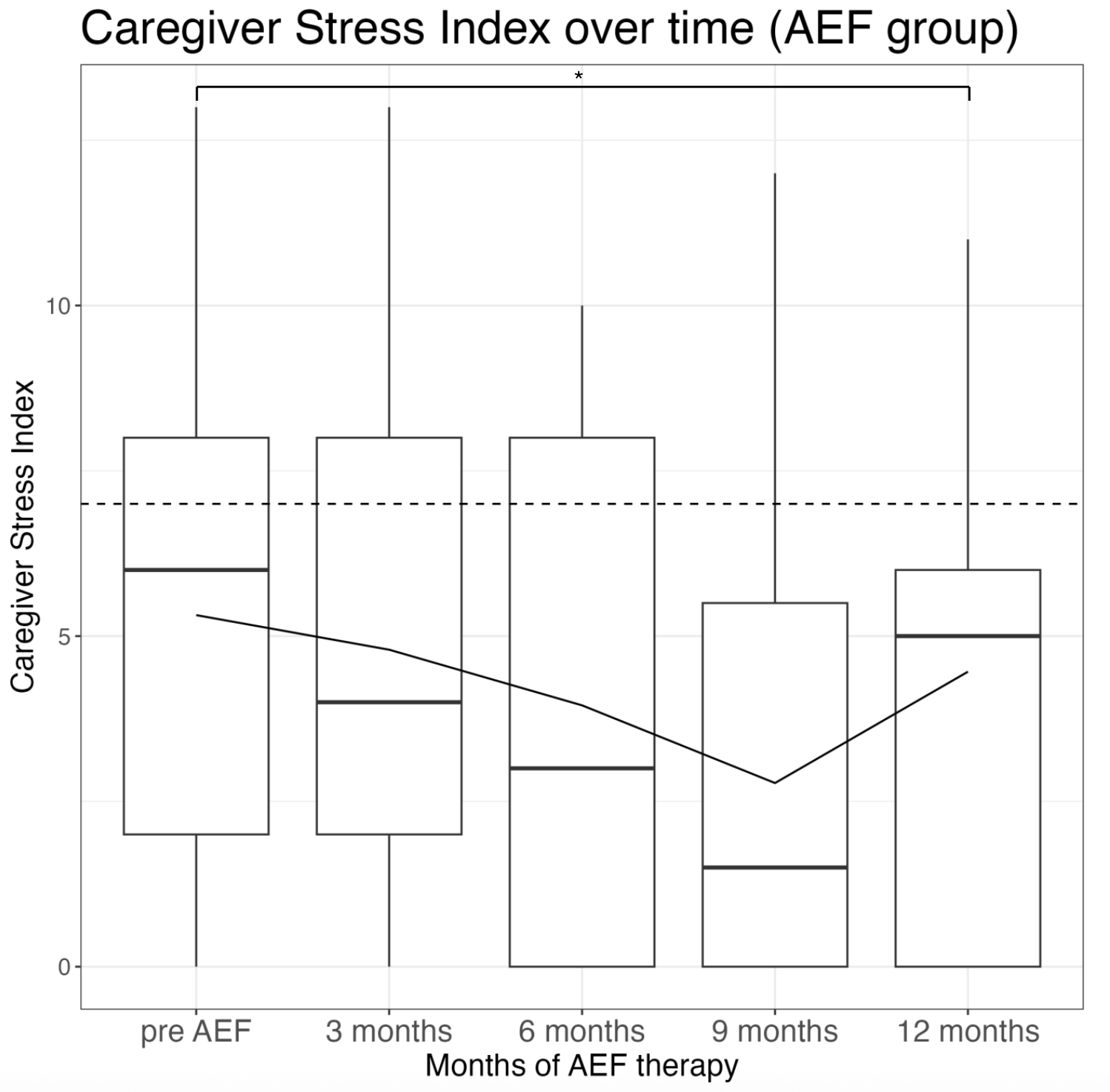

Supplement: Supplementary file 1 — Supplementary Material 1 [file 11060_2026_5631_MOESM1_ESM.tiff]
