# Supplementary material for: Health-related quality-of-life of WHO grade 4 astrocytoma patients receiving alternating electrical field therapy: a prospective real-world multi-centre study
Source: J Neurooncol. 2026 May 21;178(1):16. doi: 10.1007/s11060-026-05631-2 (PMC13194222; doi:10.1007/s11060-026-05631-2)
Supplement: Supplementary file 2 — Supplementary Material 2 [file 11060_2026_5631_MOESM2_ESM.docx]

Caregiver stress of AEF + CRT patients over time. Horizontal dotted reference line at CSI = 7, i.e. high stress. AEF: alternating electric fields. *p-value < 0.05.
